# *Saccharomyces cerevisiae*–based probiotic as novel anti-microbial agent for therapy of bacterial vaginosis

**DOI:** 10.1080/21505594.2018.1464362

**Published:** 2018-05-29

**Authors:** Samuele Sabbatini, Claudia Monari, Nathalie Ballet, Paolo Mosci, Amélie Cayzeele Decherf, Fanny Pélerin, Stefano Perito, Paolo Scarpelli, Anna Vecchiarelli

**Affiliations:** a Department of Medicine, Medical Microbiology Section, University of Perugia, Perugia, Italy; b Department of Experimental Medicine, University of Perugia, Perugia, Italy; c Lesaffre International, Lesaffre Group, Marcq-en-Baroeul, France; d Internal Medicine, Department of Veterinary Medicine, University of Perugia, Perugia, Italy; e Lesaffre Human Care, Lesaffre Group, Marcq-en-Baroeul, France

**Keywords:** probiotic, *G. vaginalis*, *S. cerevisiae*, bacterial vaginosis, sialidase, exfoliation, adherence, displacement

## Abstract

In this study, we demonstrate, for the first time, that *Saccharomyces cerevisiae*-based probiotic shows an inhibitory effect on *Gardnerella vaginalis* infection. This effect is likely due to several actions: direct interference with adherence to vaginal tissues, inhibition of sialidase activity, reduction of vaginal epithelial exfoliation. *Gardnerella vaginalis* does not induce vaginal inflammation and no inflammatory cytokines were, indeed, produced, by the mouse vagina, neither by *Gardnerella vaginalis* and by the probiotic. Collectively, our data incite to further investigations on *Saccharomyces cerevisiae* probiotic as a potential prophylactic or therapeutic agent in the vaginosis caused by *Gardnerella vaginalis*.

## Introduction

Bacterial vaginosis (BV) is the most common vaginal dysbiosis in women of childbearing age [1]. It has been associated with serious health troubles including spontaneous abortion [2], pre-term birth [3], pelvic inflammatory disease [4], endometritis [5] and enhanced acquisition and transmission of some sexually transmitted agents [6] such as HIV [7].

The clinical symptoms of bacterial vaginosis (BV) include profuse vaginal discharge and a rotten fish vaginal odor. Nevertheless many women with BV remain asymptomatic [8]. This condition is, usually, associated with dramatic reduction of healthy vaginal microflora, constituted mainly by lactobacilli, particularly *L. crispatus*, *L. jensenii* and *L. gasseri* [9,10], related to simultaneous proliferation of anaerobic bacteria including *Gardnerella vaginalis* (*G. vaginalis*), *Prevotella spp., Atopobium vaginae (A. vaginae), Bacteroides spp.* and *Mobiluncus spp* [11]. Given the high prevalence and the associated complications, BV represents an important public health issue. However, its etiology remains, yet, unclear because of great complexity and diversity of microorganisms involved [12].

Compelling evidence shows that, among bacterial multispecies involved in BV, *G. vaginalis* represents a core pathogen [13]. There is consensus that BV involves the presence of a polymicrobial structured biofilm, mainly constituted by *G. vaginalis*, strongly adhered to vaginal epithelium [14,15]. Other features associated to persistent *G. vaginalis* adherence to epithelial vaginal cells, include the activity of sialidase [15], an enzyme that plays a role in the pathogenic process, and a robust epithelial exfoliation (reminiscent of clue cells). To date therapeutic strategies, available for BV, are related to antibiotic treatment with metronidazole, clindamycin or tinidazole. Metronidazole is considered the drug of choice [16]. However very high BV recurrence rates have been reported [14,17] thus highlighting that standard antibiotic therapy was not able, in many cases, to fully eradicate BV vaginal biofilms [18,19]. It is reported [14,19,20] that antibiotic resistance, biofilm-associated, is probably a major cause of treatment failure. Furthermore, the antibiotic administration may, also, cause a dysbiosis in the vaginal flora [14]. Thus, an additional or alternative therapeutic approach, which aims to restoring the healthy vaginal microbiota, is represented by the administration of probiotics, i.e. live microorganisms providing health benefits to the host [21]. Probiotics can interfere with metabolic processes of pathogens conferring some type of protection [9,22,23]. The strains mainly used as probiotics are part of the following genera: *Bifidobacterium, Lactobacillus* and *Saccharomyces* [24]. Many studies have been performed by using probiotic *Saccharomyces cerevisiae (S. cerevisiae)* strains on gastrointestinal tract infections, where the microbial population imbalance is evident [25]. Furthermore, it has been reported that *S. cerevisiae* is able to enhance the survival and therapeutic potential of probiotic *L. rhamnosus* [25] that is, usually, used to prevent and treat vaginal infection [26]. Recently, our group demonstrated that vaginal administration of probiotic *S. cerevisiae* yeast (GI) exerted beneficial therapeutic effects on vaginal candidosis [27].

The objective of the present study was to assess the ability of *S. cerevisiae*-based probiotic to control *G. vaginalis* in an experimental mouse model of vaginal infection. We, also, addressed possible mechanisms explaining the probiotic preventive and therapeutic potential.

## Results

### Beneficial effect of *S. cerevisiae* treatment on *G. vaginalis* infection

**Figure 1.**
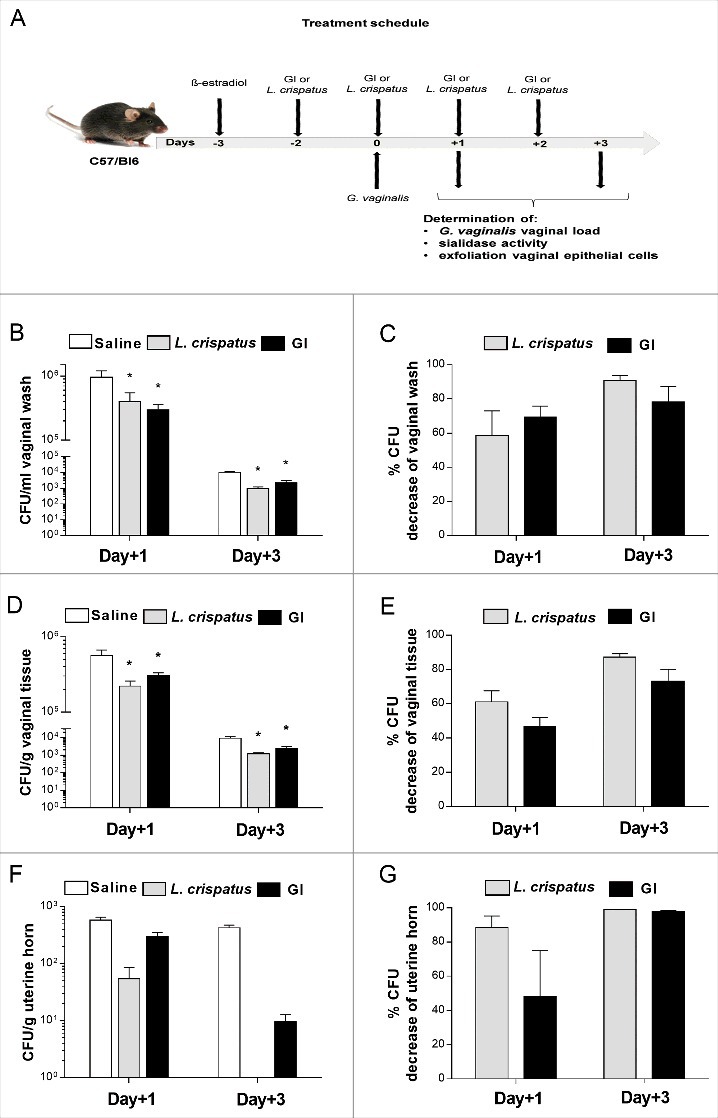
Effect of GI treatment on *G. vaginalis* infection. C57/Bl6 mice, under pseudoestrus condition, were treated intravaginally with 10 μl of Saline, or *L. crispatus* (2 × 10^9^/ml) or GI (10^8^/ml) two days before the challenge with *G. vaginalis* (5 × 10^7^/20μl/mouse) and once a day for 3 days beginning the day of infection (A). *G. vaginalis* load were determined by enumerating colony forming units (CFU) in vaginal washes (B), in tissue (D) and uterine horn homogenates (F) at days 1 and 3 post-infection. Percentage of *G. vaginalis* CFU decrease (C, E, G) was quantified relative to *G. vaginalis*-infected mice treated with Saline. Data are the mean ± SEM from 2 independent experiments each with 6 mice/group. *p < 0.05 *L. crispatus*- or GI-treated mice *vs* Saline-treated mice.

To analyze whether live *S. cerevisiae* yeast (encoded GI) was able to affect *G. vaginalis* growth in the mouse vagina, C57/Bl6 mice were treated with 0.5 mg/100 μl/mouse of ß-estradiol, three days prior to and on the day of intravaginal challenge with *G. vaginalis*. GI (10^8^ or 10^9^/ml) was administered intravaginally (10 μl/mouse) two days before challenge and every day, post-infection, until the end of experiment. Saline and *L. crispatus* (2 × 10^9^ or 2 × 10^10^/ml), both 10 μl/mouse [28], were used, respectively, as negative and positive control [29–31]. After 1 and 3 days post-infection we determined *G. vaginalis* load in vaginal washes, vaginal tissue and uterine horns. The experimental model is outlined in Fig. 1A. The results, reported in Fig. 1B, show that GI at the dose of 10 mg/ml, was able to significantly decrease the *G. vaginalis* load in vaginal washes, both 1 and 3 days post challenge. GI treatment decreased of 70% and 80% of *G. vaginalis* CFU, 1 day and 3 days post infection, respectively (Fig. 1C). Furthemore, the reduction of CFU, 1 day after challenge, occurred in 83.3% of GI treated mice and in 66.6% of *L. crispatus* treated mice. This reduction was manifested in 100% of treated mice, 3 days after challenge with both treatment, GI and *L. crispatus*.

*G. vaginalis* growth inhibition by GI was also detected in the vaginal tissue, at both days post-infection, as shown in Fig. 1, panels D, E. As for the vaginal washes, the highest inhibitory activity by GI against *G. vaginalis* in the vaginal tissue was observed 3 days after infection. In addition, the reduction of CFU tissue levels was observed in 100% of treated mice with GI as well as with *L. crispatus*, both 1 and 3 days post-infection. The higher dose of GI (10^9^/ml) produced similar effects in clearing *G. vaginalis*, suggesting that 10^8^/ml was sufficient for fighting experimental *G. vaginalis* infection (Supplementary Fig. 1A-D).

Given that *G. vaginalis* can cause ascending infections [13] we performed selected experiments to determine whether GI treatment could inhibit the colonization of uterine horns. As reported in Fig. 1F, a significant decrease of *G. vaginalis* load in uterine horns was observed. In particular (Fig. 1G) 1 day post-infection, the inhibition reached 50% after GI treatment. Three days after challenge *G. vaginalis* had been, almost, completely cleared, in all mice.

### Effect of *S. cerevisiae* treatment on sialidase activity of *G. vaginalis*

**Figure 2.**
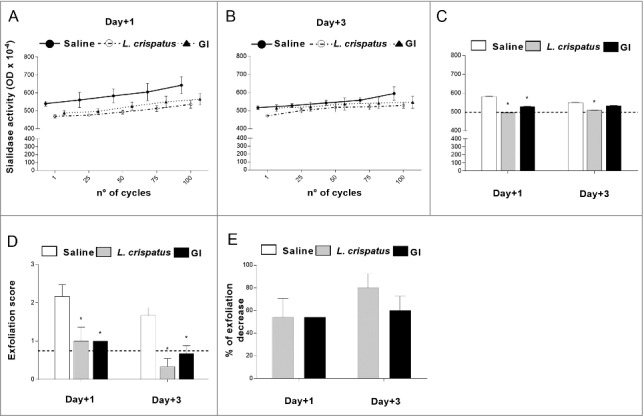
Effect of GI treatment on *G. vaginalis* sialidase activity and epithelial exfoliation *G. vaginalis*-induced. Sialidase activity and epithelial exfoliation were determined in vaginal washes of mice, treated intravaginally with 10 μl of Saline, or *L. crispatus* (2 × 10^9^/ml) or GI (10^8^/ml) and infected with *G. vaginalis* (5 × 10^7^ /20 μl/mouse) as described in Materials and Methods, at days +1 and +3 post-infection. (A, B, C) Optical density, of sialidase activity, was determined as described in Materials and Methods. (A, B) Lines are representative of experiments (n = 2) with similar results. (C) Bars are the mean ± SEM from 2 independent experiments each with 6 mice/group. The dashed line represents the optical density of sialidase activity from vaginal washes of not-infected mice. *p < 0.05 *L. crispatus*- or GI-treated mice *vs* Saline-treated mice. (D) Epithelial exfoliation score has been evaluated by assigning a value from 0 to 3, with 0 = cells number < 25 and 3 = cells number > 75. The dashed line represents the exfoliation score from vaginal washes of not-infected mice. Data are the mean ± SEM from 2 independent experiments each with 6 mice/group. *p < 0.05 *L. crispatus*- or GI-treated mice *vs* Saline-treated mice. (E) Percentage of epithelial exfoliation decrease was quantified in respect to mice treated with Saline.

The sialidase production has been associated to bacterial pathogenesis and represents a virulence factor for several pathogens such as *P. aeruginosa* [32], *V. colerae* [33], *S. pneumoniae* [34]. Since the clinical isolate of *G. vaginalis* used in our experimental model produced sialidase, we tested whether our probiotic could influence this enzymatic activity. To this purpose vaginal washes, collected from mice at days 1 and 3 post-infection, were assayed for sialidase activity (Fig. 2A–C). The results obtained show that both GI and *L. crispatus* were able to significantly inhibit this enzymatic activity particularly at day 1 post-infection (Fig. 2C). The administration of probiotics alone, without infection, did not produce detectable sialidase activity.

### Effect of *S. cerevisiae* treatment on epithelial exfoliation *G. vaginalis*-induced

It has been reported that the clue cells, which are one of the key cytological features of *G. vaginalis*-induced BV, are the result of exfoliation of vaginal epithelium [13]. Enzymes, such as sialidase, and organic acid, produced by anaerobic microorganisms, are the potential cause of exfoliation [13]. We therefore asked whether, in our experimental model, GI treatment was able to affect the epithelial exfoliation due to *G. vaginalis* infection. To give a semi-quantitative perspective of this effect, we scored the degree of exfoliation with 0 being none and 3 being very robust degree of exfoliation.

As shown in Fig. 2D, GI, as well as, *L. crispatus* were able to significantly inhibit the *G. vaginalis*-induced epithelial exfoliation. This effect was, already, evident 1 day post-infection when GI, inhibited more than 50% of exfoliation process (Fig. 2E) and remained constant over the experimental time period. The administration of probiotics alone, without infection, did not produce detectable exfoliation.

### Effect of *S. cerevisiae* treatment on immune vaginal response

**Figure 3.**
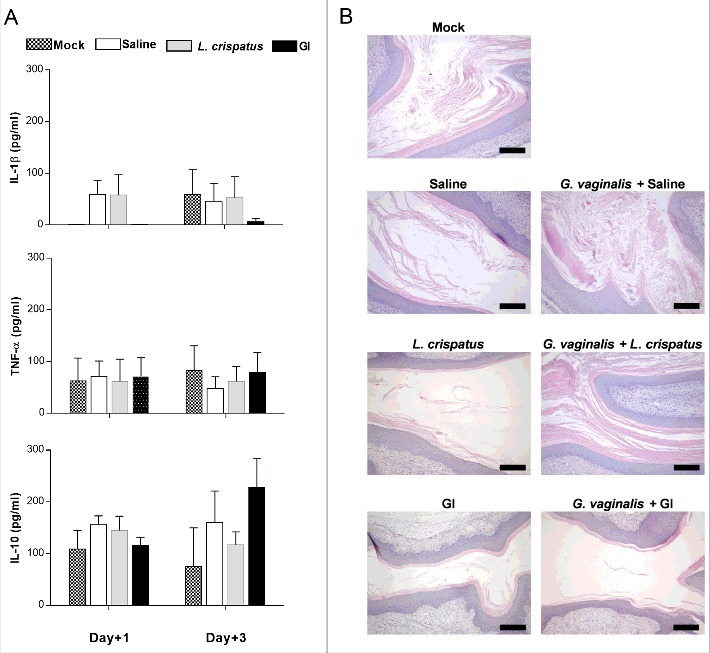
Evaluation of immune response to probiotic treatment. (A) Pro-(IL-1β and TNF-α) and anti-(IL-10) inflammatory cytokines levels have been determined in vaginal washes of mice, treated intravaginally with 10 μl of Saline, or *L. crispatus* (2 × 10^9^/ml) or GI (10^8^/ml) and infected with *G. vaginalis* (5 × 10^7^/20 μl/mouse), at days +1 and +3 post-infection. Data are the mean ± SEM from 2 independent experiments each with 6 mice/group. (B) Histological inflammation was assessed by haematoxylin-eosin staining of formalin-fixed, paraffin-embedded vaginal tissue sections. Images (Bar = 200 µm, Magnification 10x) are representative of 2 separate experiments with similar results.

To verify whether the clearance of *G. vaginalis* by probiotics was associated to any stimulation of immune response, the possibility that probiotics could affect the cytokine secretion in the local vaginal area was evaluated. To this end pro-inflammatory (IL-1ß and TNF-α) and anti-inflammatory (IL-10) cytokines were determined in vaginal washes at day+1 and +3 post-infection. The results show that no significant variations of cytokine levels was observed after infection with *G. vaginalis* respect to saline treated mice. The treatment with probiotics did not alter this condition (Fig. 3A). Moreover, histological analysis of vaginal tissue, from mice treated with probiotics alone, or infected and treated with probiotics, shows that no inflammatory cells were present in vaginal tissue in any of the histological preparations. These results confirm that, at variance with other vaginal infections [27], there is no inflammatory response in the vaginal tissue of mice challenged with *Gardnerella* and treated with probiotics.

### *S. cerevisiae* inhibits *G. vaginalis* adherence on vaginal and cervix epithelial cells

**Figure 4.**
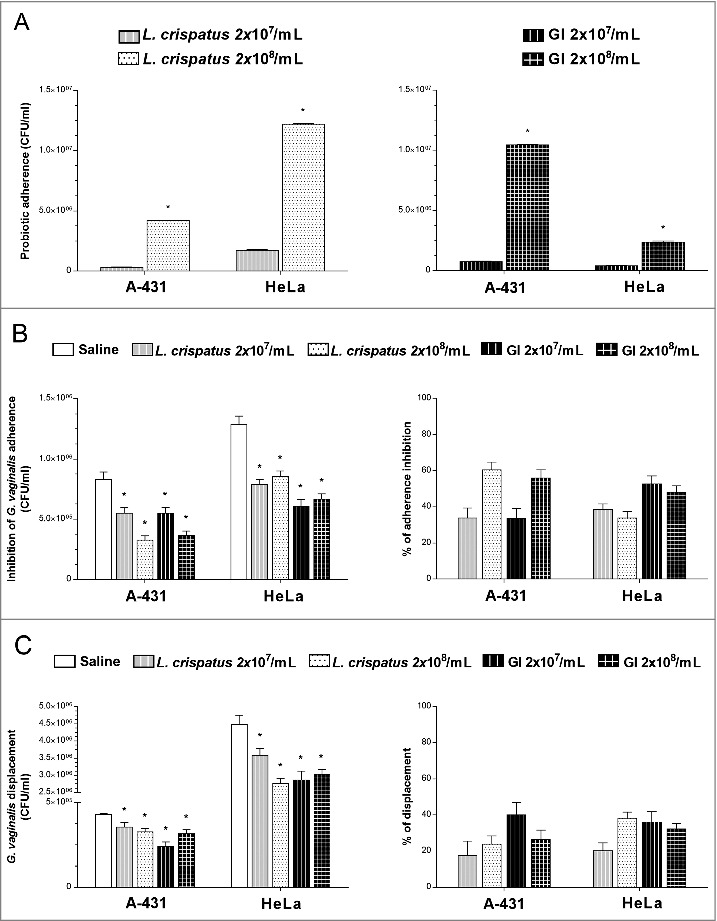
Effect of GI treatment on *G. vaginalis* adherence to vaginal (A-431) and cervix (HeLa) epithelial cells. (A) Adhesion of *L. crispatus* or GI to A-431 and to HeLa cell lines. *L. crispatus* or GI (both 2 × 10^7^/ml or 2 × 10^8^/ml) were added to monolayer of A-431 or HeLa cells for 4 h at 37°C in anaerobic conditions. After incubation, cells were washed 2 times and microorganisms adhered were quantified as number of CFU/ml. Data are the mean ± SEM from 2 independent experiments. *p < 0.05, 2 × 10^8^/ml (*L. crispatus* or GI) *vs* 2 × 10^7^/ml (*L. crispatus* or GI). (B) Interference of *L. crispatus* or GI on *G. vaginalis* initial adhesion onto A-431 and HeLa cell lines. Two inocula (2 × 10^7^/ml or 2 × 10^8^/ml) of *L. crispatus* or GI were pre-adhered to epithelial cells, as above described, and subsequently *G. vaginalis* (2 × 10^8^/ml) has been added to the co-culture for 30 min at 37°C in anaerobic conditions. *G. vaginalis* adhered were quantified as number of CFU/ml. *p < 0.05 *L. crispatus* or GI *vs* Saline treatment. Percentage adherence inhibition was quantified in respect to Saline. (C) Reduction of *G. vaginalis* adherent to epithelial cells. *G. vaginalis* (2 × 10^8^/ml) was incubated with the monolayers for 30 min at 37°C in anaerobic conditions. Then, non-adherent bacteria were removed by washing and probiotics (2 × 10^7^/ml or 2 × 10^8^/ml) were added to co-cultures for 30 min at 37°C in anaerobic conditions. *G. vaginalis* displacement was expressed as CFU/ml as described in Material and Methods. *p < 0.05 *L. crispatus* or GI *vs* Saline treatment.

Given that immune cells do not play a role in the GI induced *G. vaginalis* clearance, we evaluated whether some other mechanistic effects were involved in the inhibition of *G. vaginalis* load. Indeed, adhesion to host cells is a critical initial step in any infectious process and *in vitro* models of infections have been extensively used for analyzing the interactions between non-pathogenic and pathogenic bacteria [27,29,35–37]. Therefore, we investigated whether GI was able to inhibit the *G. vaginalis* adhesion on epithelial cells by using an *in vitro* model system such as vaginal (A-431) and cervix (HeLa) epithelial cell lines. In a first series of experiments, we analyzed the capacity of GI and *L. crispatus* (each at two different doses) [29,31], to adhere to A-431 or HeLa cells. To this end, the cells were treated with GI or *L. crispatus* and, after extensive washings, colony forming units (CFU) were determined. The results reported in Fig. 4A show that both GI and *L. crispatus* were able to adhere, with different degrees, to vaginal and cervix epithelial cells. GI manifested a better capacity to adhere to A-431 cells than to HeLa cells, whereas *L. crispatus* showed an opposite behavior. Furthermore this interaction occurred in a dose dependent manner. Then the capacity of GI to compete for *G. vaginalis* adhesion on A-431 and HeLa cells was determined. A-431 and HeLa cells were treated with GI or *L. crispatus* (each at two different doses) for 4h at 37°C and, after extensive washing, *G. vaginalis* was added. The control was *G. vaginalis* adhesion only. The results reported in Fig. 4B left panel, show that a significant inhibition of *G. vaginalis* adhesion to A-431 and HeLa cells was observed with both doses of GI used. The inhibition of adherence reached about 40–50% for both A-431 and HeLa cells. Similar results were obtained by using of *L. crispatus* (Fig. 4B right panel).

### *S. cerevisiae* induces the displacement of *G. vaginalis* adhered on vaginal and cervix epithelial cells

The capacity of GI to inhibit *G. vaginalis* adherence suggested the further possibility that GI could exert a displacement of pre-adhered *G. vaginalis* to epithelial cells. To this end the epithelial cells were treated with *G. vaginalis* and, after extensive washings to remove non adherent bacteria, GI or *L. crispatus* were added. The results reported in Fig. 4C left panel, show that a consistent amount of *G. vaginalis* was removed by GI. *L. crispatus* showed similar effect. Both doses of GI were effective in displacing *G. vaginalis* from epithelial and cervix vaginal cells. (Fig. 4C, right panel).

### *S. cerevisiae* does not induce *G. vaginalis* co-aggregation

**Figure 5.**
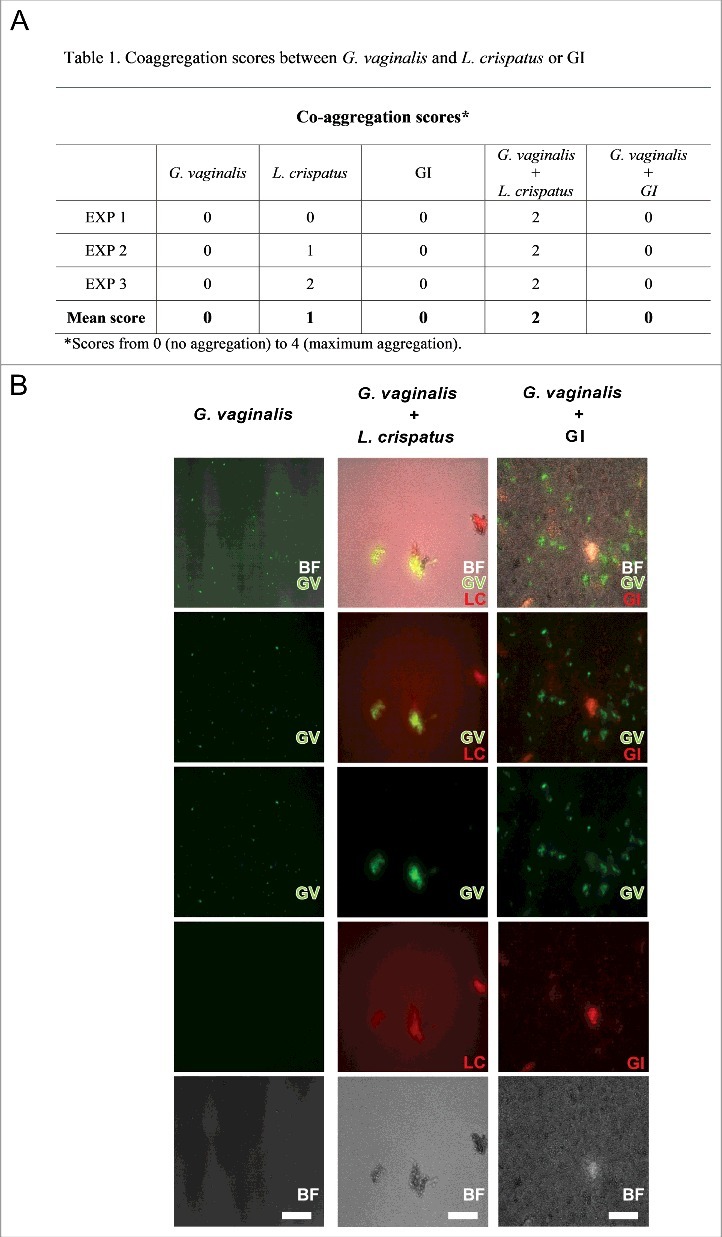
Co-aggregation between GI or *L. crispatus* and *G. vaginalis*. *G. vaginalis* or FITC-*G. vaginalis* (1 × 10^9^/ml) in PBS were mixed with equal volume of *L. crispatus* or RhB-*L. crispatus* (1 × 10^9^/ml) or with equal volume of GI or RhB-GI (10^8^/ml). The samples were vortexed for at least 10 sec and incubated in a 24 well plate for 4 h at 37°C under agitation. The suspensions were, then, observed by inversion light microscopy to evaluate the aggregation degree or photographed by fluorescence microscopy. (A) Scores, from 0 (no aggregation) to 4 (maximum aggregation), and mean are shown. Data are from replicate samples of 3 different experiments. (B) Images are representative of 3 different experiments with similar results (Scale Bar = 50 µm, Magnification 20x). BF = bright field; *G. vaginalis* (GV) = green; *L. crispatus* (LC) = red.

Another important mechanistic effect for eliminating bacteria is the capacity to aggregate pathogens. Indeed, co-aggregation is one of the mechanisms exerted by probiotics to create a competitive micro-environment around the pathogen [38,39]. In this line, GI was tested for its capacity to co-aggregate with *G. vaginalis*. To this purpose GI was incubated alone or mixed with *G. vaginalis*. Neither *G. vaginalis* nor GI self-aggregated at any of the tested doses. Results of co-aggregation showed that GI was unable to co-aggregate with *G. vaginalis*. Conversely *L. crispatus* was able to self-aggregate and induced co-aggregation of *G. vaginalis* (Fig. 5A). A representative image, demonstrating that GI was not able to self-aggregate as well as to co-aggregate *G. vaginalis* is reported in Fig. 5B.

## Discussion

Bacterial vaginosis is a polymicrobial clinical syndrome in which *Lactobacillus spp.*, major constituents of “normal vaginal microbiota”, are replaced by an overgrowth of non-beneficial anaerobic microbial species. This dysbiosis is recognized as the most common cause of abnormal vaginal discharge in women of childbearing age and it is associated with serious pregnancy-related sequelae and increased transmission of sexually transmissible infections. *G. vaginalis* is the most frequent microorganism isolated from vaginal fluids of women suffering from BV [40,41].

Many studies have suggested that the presence of vaginal lactobacilli may protect against BV [31,39,42–45]. The dominant *Lactobacillus spp.* include *L. crispatus, L. gasseri* and *L. jensenii* [46]. There is general consensus that *L. crispatus* inhibits *G. vaginalis* growth by producing lactic acid [31] and additional studies provide evidence for inhibition of *G. vaginalis* adherence to host cells [29,39,45,47]. Women colonized by *L. crispatus* show a decreased risk of developing BV [30]. For all these reasons *L. crispatus* was included in our experimental system as positive control.

**Figure 6.**
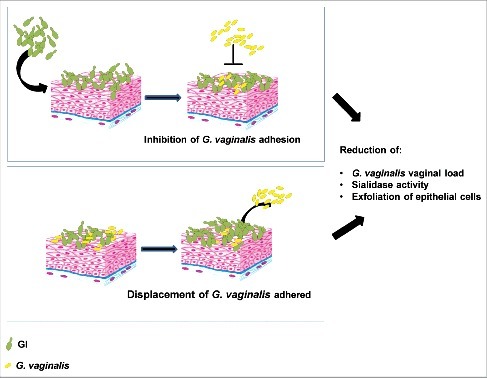
Schematic representation of GI mechanism on *G. vaginalis* infection. GI, by inhibition of *G. vaginalis* adhesion and by displacement of *G. vaginalis* adhered to epithelial cells, reduces *G. vaginalis* vaginal load and its key virulence factors.

Here we demonstrate, for the first time, that a *S. cerevisiae*-based probiotic shows a marked antagonistic effect against *G. vaginalis* colonization in vaginal environment and preclude the access of *G. vaginalis* to uterine horns. This is associated with inhibition of important virulence factor such as sialidase activity, with decreased exfoliation of vaginal epithelial cells and decreased adherence to them in model systems. Indeed, GI is able to adhere to vaginal epithelial cells and by these specific traits allows the inhibition of *G. vaginalis* adherence to EC. Nevertheless, GI not only inhibits *G. vaginalis* adhesion, but it is also able to displace *G. vaginalis* attached to EC (see mechanism of action in Fig. 6). It is well known that *L. crispatus* is able to inhibit the growth of *G. vaginalis*, however *S. cerevisiae*, also effective against vaginal candidosis, would represent an additional therapeutic option for preventing or curing vaginal infections.

We previously reported that the treatment with GI is beneficial during vaginal candidosis [27] and in this study we demonstrate that GI is also able to antagonize *G. vaginalis* infection. Indeed, by using a well-known *in vivo* mouse experimental model [13], we showed that intravaginal administration of *S. cerevisiae*-based probiotic (10^8^/ml) was able to remove, 3 days after infection, about 80% of *G. vaginalis* from the mouse vagina in all the treated mice. A higher dose of probiotic (10^9^/ml) was not more effective than the lower one reported above. Notably, no intervention of a local immune response appears to be present in the vaginal infection by *G. vaginalis*, confirming previous results [13]. This is in clear contrast with *C. albicans* infection [27], as clinically documented, and justifying the use of the mouse model as a useful simulator of human infection.

Reports have identified *G. vaginalis* as an etiologic agent in puerperal sepsis [48,49], endometritis and septic abortion [50]. The pathogenesis of these infections is considered to be a consequence of the microorganism spread from the vagina to the uterus and urogenital tract, due to mucosal damage during delivery. With this scientific background we determined if GI treatment affected *G. vaginalis* infection at the level of uterine horns. Indeed, GI significantly reduces the bacterial load and 3 days after infection, it was able to remove up to 90% of *G. vaginalis* infecting uterine horns. These results consistently demonstrate that GI presents a potential beneficial effect not only in vaginal infections but, also, in ascending infections and its potentially dramatic effects.

Previous investigations have shown that, in the vaginal fluid of BV patients, the levels of sialidase activity were increased compared to those detected in women with normal flora [51,52]. In addition, Gilbert *et al.* [13], in an *in vivo* experimental model, reported that the level of sialidase activity correlated with vaginal *G. vaginalis* titers. In our experimental model GI markedly reduced sialidase activity thereby reducing *G. vaginalis* virulence. Given that sialidase is an enzyme known to facilitate the destruction of the protective mucus layer on the vaginal epithelium [53] it is conceivable that GI exerts a protective effect from BV. This inhibitory effect could be due to production of GI soluble factors that degrade the enzyme and/or to direct inhibition of gene expression. These results, also, suggest that modulation of sialidase expression, by the use of appropriate probiotics or specific inhibitors could be exploited for therapeutic purposes.

A key feature used to diagnose BV is the formation of clue cells which are the result of exfoliation of vaginal epithelium. A recent paper reports that vaginal epithelial cells exfoliation occurs in an experimental model of *G. vaginalis* infection and in clinical specimens from women with BV [13]. It is conceivable that a poor exfoliation could be beneficial in eliminating a potential pathogen, whereas a marked exfoliation could facilitate the pathogen diffusion through adhesion to underlying tissues. In our experimental model GI strongly reduces the exfoliation induced by *G. vaginalis* infection likely avoiding the pathogens spread and adhesion to internal tissues.

Altogether, our data clearly demonstrate that GI has a strong capacity to fight *G. vaginalis* experimental infection, that its efficiency is comparable to that of *L. crispatus*, recognized probiotic in the treatment of BV, and that several mechanisms can contribute to this beneficial effect. The probiotic capacity, reported here, to displace adherent *G. vaginalis* from epithelial vaginal cells and epithelial cervix cells is of special interest for potential therapeutic purposes in humans. However we cannot exclude that other mechanisms, generated by cell-cell contact, could interfere with expression of virulence gene and/or affecting the growth conditions. To our knowledge this is the first report demonstrating that *S. cerevisiae*-based probiotic can exert an inhibitory effect on *G. vaginalis* infection. Collectively our data suggest the potential use of *S. cerevisiae*-based probiotic for the prophylaxis and/or treatment of bacterial vaginosis. Our results strongly encourage further studies about the capacity of this probiotic to prevent and manage urogenital tract infections in women.

## Materials and methods

### Study products

The product studied was provided by Lesaffre Human Care (Marcq-en-Baroeul, France). *Saccharomyces cerevisiae* (*S. cerevisiae*) live yeast (referenced GI) is a proprietary, well-characterized strain of Lesaffre, registered in the French National Collection of Cultures of Microorganisms (CNCM) under the number I-3856. The *S. cerevisiae* species was determined by using phenotypic (API®ID32C, Biomerieux SAS) and genotypic referenced methods (genetic amplification and sequencing of 26S DNA) [54,55]. Moreover, the strain CNCM I-3856 has been characterized by polymerase chain reaction (PCR) Interdelta typing techniques [56] and other genetic methods (*e.g*., complete genome sequencing).

The specification of the probiotic product is ≥5 × 10^9^ CFU/g and the concentration of the batch used for these trials was 1 × 10^10^ CFU/g.

The strain of *L. crispatus* 33820, used in this study, was obtained from the American Type Culture Collection (ATCC).

### Microbial strains and growth conditions

Sialidase-positive *G. vaginalis* clinical isolate was obtained from a vaginal swab from the Microbiology Unit of Santa Maria della Misericordia Hospital of Perugia. The swab was immediately used to inoculate Gardnerella selective agar (GSA) media (plates with 5% of human blood, Becton and Dickinson) and the plates were incubated anaerobically at 37°C for 24–48 hours. ß-haemolytic colonies were isolated and candidate *G. vaginalis* strains were identified by Matrix-Assisted Laser Desorption/Ionization Time-of-Flight (MALDI-TOF, Bruker Daltonics) mass spectrometry. A spontaneous streptomycin-resistant mutant was isolated by plating *G. vaginalis* on New York City III (NYC-III) agar plates +1 mg/ml streptomycin and selecting resistant colonies after incubating anaerobically at 37°C for 72 hours. Results for sialidase activity and growth curves of resistant mutant were indistinguishable from those of the clinical isolate. The *G. vaginalis* resistant mutant has been used for both our *in vivo* and *in vitro* experimental models. *L. crispatus* ATCC 33820 was grown anaerobically in de Man, Rogosa and Sharpe broth (MRS, Sigma). Before each experiment the strains were harvested by centrifugation for 5 min at 11000 rpm, washed twice with sterile phosphate-buffered saline (PBS, Life Technologies), the concentration adjusted to that desired and resuspended in the appropriate buffer.

### Ethics statement

The procedures involving the animals and their care were conducted in conformity with the national and international laws and policies. All animal experiments were performed in agreement with the EU Directive 2010/63, the European Convention for the Protection of Vertebrate Animals used for Experimental and other Scientific Purposes, and the National Law 116/92. The protocol was approved by Perugia University Ethics Committee for animal care and use (Comitato Universitario di Bioetica, permit number 308/2017-PR). All the animals were housed in the animal facility of the University of Perugia (Authorization number 34/2003A). Mice were acclimatized for a week before starting the experiments. 6 mice were housed in each cage and were provided with food and water *ad libitum*. All efforts were made to minimize suffering during experiments.

### Mice

Female C57/Bl6 mice obtained from Charles River (Calco, Italy) and acclimatized for 1 week before starting experiments were used at 5 to 7 weeks of age. Animals were used under specific-pathogen free conditions that included testing sentinels for unwanted infections. According to the Federation of European Laboratory Animal Science Association standards, no infections were detected.

### Culture of A-431 and HeLa cell lines

A-431 (ATCC CRL-1555) and HeLa epithelial cells (ATCC CCL-2) were cultured, at 37°C and in 5% CO_2_, in DMEM supplemented with 15% (vol/vol) fetal bovine serum (FBS, Life Technologies) and 1 IU penicillin/streptomycin ml^−1^ (Lonza). Cells were cultured, at 37°C and 5% CO_2,_ in 24-well tissue culture plates (Iwaki) until they formed a monolayer. Before the adhesion assays, the cells were washed twice with 500 μl of sterile phosphate -buffered saline (PBS) to remove non adherent cells and culture media.

### *G. vaginalis* infection model

A mouse model of *G. vaginalis* infection was previously described by Gilbert *et al.* [13].Mice were injected with 0.5 mg ß-estradiol in 100 μl sesame oil three days prior to and on the day of infection. A suspension of ∼ 5 × 10^7^ CFU of *G. vaginalis* in 20 μl of sterile PBS was vaginally inoculated in mice anaesthetized with isoflurane. GI (10^8^/ml = 10 mg/ml or 10^9^/ml = 100 mg/ml) or *L. crispatus* (2 × 10^9^/ml = 10 mg/ml or 2 × 10^10^/ml = 100 mg/ml) were administered intravaginally (10 μl/mouse) two days before challenge and once a day for 3 days beginning the day of infection.

At days 1 and 3 post-infection, the mice were sacrificed and vaginal washes were collected by flushing vaginas with sterile physiological solution. The fluid was serially diluted and plated on NYC-III agar plates +1 mg/ml streptomycin and 4 mg/L amphotericin. Colonies were, then, enumerated and expressed as CFU/ml. The percentage of CFU reduction, as consequence of treatment with probiotics, was determined by subtracting the *G. vaginalis* CFU of probiotics-treated mice from *G. vaginalis* CFU of saline-treated mice and expressed as the percentage of CFU decrease. Vaginal washes were, also, tested for sialidase activity, epithelial exfoliation and cytokines levels as described below.

In selected experiments at days 1 and 3 post-infection, the mice were sacrificed and half of the vaginas and one uterine horn from each mouse were harvested, homogenized and plated on NYC-III agar plates +1 mg/ml streptomycin and 4 mg/L amphotericin for CFU evaluation as for vaginal washes. The remaining vaginal tissue were fixed in 10% buffered formalin phosphate, embedded in paraffin, sectioned into 3 to 4 µm thick sections, and stained with H&E.

### Sialidase activity assay

Sialidase activity was assessed in vaginal wash samples. Briefly, 50 μl of each vaginal wash were diluted 1:1 with working solution of Amplex Red Neuraminidase (Sialidase) Assay Kit (Thermo) and incubated at 37°C. The kinetics of the reactions were followed by measuring absorbance at 560 nm at multiple time points using a Tecan Infinite M200 plate reader.

### Epithelial cell exfoliation

To assess the exfoliation of mouse vaginal epithelium, wet mounts were prepared with 5 µl of vaginal wash and visualized by phase contrast microscopy using Olympus KX31 microscope. Samples score was assigned from 0 to 3 depending on the average number of epithelial cells in microscope fields: 0 = cells number <25, 1 = cells number from 25 to 50, 2 = cells number from 50 to 75 and 3 = cells number >75 [13].

### Cytokines

Supernatants of vaginal washes were collected and tested for Interleukin-1ß (IL-1ß), TNF-α, IL-6 and IL-10 levels by specific ELISAs (Thermo Fisher Scientific). Cytokine titers were calculated relative to standard curves.

### Adhesion and displacement assays

Two distinct experiments were performed to study the influence of probiotics on the adhesion mechanisms of *G. vaginalis* to epithelial cells. First, the interference of pre-adhered probiotics, on epithelial cells, towards *G. vaginalis* was evaluated. To this aim, two distinct cell quantities (2 × 10^7^/ml and 2 × 10^8^/ml) [29] of each probiotic were added to each well of the 24-well containing the monolayers. The plates were incubated for 4 h at 37°C in anaerobic conditions. Non adherent probiotics were removed by washing with 500 μl of sterile PBS (2 times) then *G. vaginalis* (2 × 10^8^/ml) [29] was incubated with the monolayers (final volume 500 µl) for 30 min at 37°C in anaerobic conditions. Each well was carefully washed (2 times) with 500 μl of sterile PBS to remove non-adherent bacteria. To evaluate CFU of adhered *G. vaginalis*, the medium was removed and Trypsin/EDTA solution (200 µl) was added in each well to dissociate cells [27]. Hence, the cellular suspension was serially diluted, plated onto NYC-III agar plates and incubated at 37°C for 48 h in anaerobic condition. The *G. vaginalis* load was quantified as the number of CFU/ml. In the second set of experiments, the ability of probiotics to displace *G. vaginalis* pre-adhered to monolayers was assessed. To this end, *G. vaginalis* (2 × 10^8^/ml) was incubated with the monolayers for 30 min at 37°C in anaerobic conditions. Wells were washed twice with 500 μl of sterile PBS to remove non adherent bacteria, then probiotics (2 × 10^7^/ml or 2 × 10^8^/ml) were added to the appropriate wells for 30 min at 37°C in anaerobic conditions. Finally, each well was washed twice with sterile PBS to remove non-adherent *G. vaginalis* and probiotics [29]. Quantification of *G. vaginalis* adherent to epithelial cells was performed as above described and expressed as CFU/ml.

### Co-aggregation assay

The co-aggregation assay was performed as previously described [57]. *G. vaginalis* cells (1 × 10^9^/ml) were labeled with Fluorescein isothiocyanate (FITC, Sigma) at 0.1 mg/ml in PBS at room temperature (RT) for 10 min. *L. crispatus* (2 × 10^9^/ml) and GI (10^8^/ml) were labeled with Rhodamine B (0.5 mg/ml, Sigma) in PBS for 20 min at RT. Briefly, *G. vaginalis* or FITC-*G. vaginalis* (1 × 10^9^/ml) in PBS were mixed with equal volume of *L. crispatus* or RhB-*L. crispatus* (2 × 10^9^/ml) or with equal volume of GI or RhB-GI (10^8^/ml). Then samples were vortexed for at least 10 sec and incubated in a 24 well plate for 4 h at 37°C under agitation. The suspensions were then observed by inversion light microscopy to evaluate the aggregation degree and scored according to the following scale: 0 = no aggregation, 1 = small aggregates comprising small visible clusters, 2 = aggregates comprising larger numbers of microorganisms, settling down to the center of the well, 3 = macroscopically visible clumps comprising larger groups which settle to the center of the well, 4 = maximum score allocated to describe a large, macroscopically visible clump in the center of the well [57]. Moreover, each fluorescent suspension was analyzed under a fluorescence microscope (Carl Zeiss).

### Statistical analysis

GraphPad Prism 7.0 software was used for all statistical analysis presented. For the analysis of sialidase activity, differences between *L. crispatus*- or GI-treated infected mice *vs* saline-treated infected mice were evaluated by Mann-Whitney U-test. For the other experiments, the results were evaluated by Student's t test. Values of *p* < 0.05 were considered significant.

## Supplementary Material

Supplementary_Figure_1.docx
