# Supplementary material for: Saccharomyces cerevisiae–based probiotic as novel anti-microbial agent for therapy of bacterial vaginosis
Source: Virulence. 2018 May 29;9(1):954–66. doi: 10.1080/21505594.2018.1464362 (PMC6037478; doi:10.1080/21505594.2018.1464362)
Supplement: Supplementary_Figure_1.docx [file kvir-09-01-1464362-s001.docx]

**Supplementary Figure 1.** Effect of GI (100 mg/ml) treatment on *G. vaginalis* infection. C57/Bl6 mice, under pseudoestrus condition, were treated intravaginally with 10 μl of Saline, or *L. crispatus* (100 mg/ml) or GI (100 mg/ml) two days before the challenge with *G. vaginalis* (5 x 10^7^ /20 μl/mouse) and once a day for 3 days beginning the day of infection. *G. vaginalis* titers were determined by enumerating colony forming units (CFU) in vaginal washes (A) and in tissue homogenates (C) at days 1 and 3 post infections. Percentage of CFU decreased (B, D) were quantified relative to *G. vaginalis* infected mice-treated with Saline. Data are the mean ± SEM from 2 independent experiments each with 6 mice/group. *P<0.05 *L. crispatus* or GI-treated mice *vs* Saline-treated mice.
